# Detection of Common Respiratory Infections, Including COVID-19, Using Consumer Wearable Devices in Health Care Workers: Prospective Model Validation Study

**DOI:** 10.2196/53716

**Published:** 2024-07-17

**Authors:** Zeinab Esmaeilpour, Aravind Natarajan, Hao-Wei Su, Anthony Faranesh, Ciaran Friel, Theodoros P Zanos, Stefani D’Angelo, Conor Heneghan

**Affiliations:** 1 Google LLC San Francisco, CA United States; 2 Northwell Health New Hyde Park, NY United States; 3 Institute of Health System Science Feinstein Institutes for Medical Research Northwell Health New York, NY United States

**Keywords:** COVID detection, wearable, respiratory virus detection, algorithm, respiratory infection, respiratory virus, COVID-19, wearable device, well-being, health, physiology, health care worker, prediction, infection, physical stress, emotional stress

## Abstract

**Background:**

The early detection of respiratory infections could improve responses against outbreaks. Wearable devices can provide insights into health and well-being using longitudinal physiological signals.

**Objective:**

The purpose of this study was to prospectively evaluate the performance of a consumer wearable physiology-based respiratory infection detection algorithm in health care workers.

**Methods:**

In this study, we evaluated the performance of a previously developed system to predict the presence of COVID-19 or other upper respiratory infections. The system generates real-time alerts using physiological signals recorded from a smartwatch. Resting heart rate, respiratory rate, and heart rate variability measured during the sleeping period were used for prediction. After baseline recordings, when participants received a notification from the system, they were required to undergo testing at a Northwell Health System site. Participants were asked to self-report any positive tests during the study. The accuracy of model prediction was evaluated using respiratory infection results (laboratory results or self-reports), and postnotification surveys were used to evaluate potential confounding factors.

**Results:**

A total of 577 participants from Northwell Health in New York were enrolled in the study between January 6, 2022, and July 20, 2022. Of these, 470 successfully completed the study, 89 did not provide sufficient physiological data to receive any prediction from the model, and 18 dropped out. Out of the 470 participants who completed the study and wore the smartwatch as required for the 16-week study duration, the algorithm generated 665 positive alerts, of which 153 (23.0%) were not acted upon to undergo testing for respiratory viruses. Across the 512 instances of positive alerts that involved a respiratory viral panel test, 63 had confirmed respiratory infection results (ie, COVID-19 or other respiratory infections detected using a polymerase chain reaction or home test) and the remaining 449 had negative upper respiratory infection test results. Across all cases, the estimated false-positive rate based on predictions per day was 2%, and the positive-predictive value ranged from 4% to 10% in this specific population, with an observed incidence rate of 198 cases per week per 100,000. Detailed examination of questionnaires filled out after receiving a positive alert revealed that physical or emotional stress events, such as intense exercise, poor sleep, stress, and excessive alcohol consumption, could cause a false-positive result.

**Conclusions:**

The real-time alerting system provides advance warning on respiratory viral infections as well as other physical or emotional stress events that could lead to physiological signal changes. This study showed the potential of wearables with embedded alerting systems to provide information on wellness measures.

## Introduction

The COVID-19 pandemic caused by the SARS-CoV-2 virus had a major impact on public health since its emergence in late 2019. The ability to provide early accurate detection of the virus has been important for controlling the spread of the virus in the community [1]. Virus transmission from asymptomatic or presymptomatic individuals has been a key factor contributing to the spread. High levels of SARS-CoV-2 virus have been observed 48-72 hours before symptom onset [2].

At present, 1 in 5 Americans use wearable devices [3]. Longitudinal information collected from fitness trackers and smartwatches holds immense potential for real-time health tracking and illness detection [4-8]. Infection detection based on physiological signals can help bridge the existing gap in the diagnosis and treatment of viral infections and intelligently provide guidance on who might be at risk for infections and hence help limit the spread. Recent studies have shown that wearable devices could detect respiratory infections such as COVID-19 and influenza [9-16]. Different combinations of physiological signals, such as resting heart rate, heart rate variability, sleep data, respiratory rate, dermal temperature, step counts, and physical activity, have been used for upper respiratory infection prediction models with promising results [10-13,17-19].

In a previous investigation by our team, a model was developed to associate changes in respiratory rate, resting heart rate, and heart rate variability (measured using trackers or smartwatches) with the onset of COVID-19 [9]. These features were combined into an “alerting” algorithm, which would indicate the day on which the subject was believed to have contracted COVID-19. The investigation noted a sensitivity of 43% and a specificity of 95% in correctly labeling days as either being associated with COVID-19 or being healthy days, using a window of 7 days after the onset of COVID-19 symptoms. However, these results were generated using a retrospective self-reported survey instrument, with no direct laboratory confirmation on the timing or accuracy of positive cases. In this prospective validation study, our primary objective was to evaluate the performance of the previously developed algorithm in real-time alerting for COVID-19 infections and our secondary objective was to evaluate the performance of the model for other upper respiratory infections in a sample of health care workers affiliated with Northwell Health in New York.

## Methods

### Participants

Northwell Health (Northwell) workforce members or affiliates (ie, students, faculty members, and staff) were invited to participate (across Northwell’s 21 hospitals, more than 850 outpatient facilities, and research institutes). Northwell has over 70,000 employees, with a significant number engaged in frontline clinical work. Participants were recruited into the trial via internal employee messaging systems and flyers, with enrollment taking place remotely via an online platform (REDCap). Before enrollment, participants underwent an eligibility screening. The inclusion criteria were as follows: (1) age of 18 years or older, (2) Northwell Health member or affiliate, (3) ability to speak or read English, (4) ability to give informed written consent, and (5) owning a smartphone capable of receiving text messages and connecting to the internet. We excluded participants who (1) were pregnant or lactating women, (2) had a pacemaker or implantable cardioverter defibrillator, or (3) were unable or unwilling to wear a device. Potential participants completed the screening and consented online via a Northwell-approved electronic data capture platform (REDCap). Initial recruitment was focused on those employees identified as being at higher risk of contracting COVID-19 (eg, nurses, doctors, and others with direct exposure to COVID-19 patients) [20]. All participants were vaccinated with at least one dose of the COVID-19 vaccine in line with the Northwell mandate for vaccination.

### Ethical Considerations

The study was approved by the Northwell Institutional Review Board (IRB#20–1080). All participants provided written informed consent under the approved protocol (IRB#20–1080), and all research procedures were performed in accordance with relevant guidelines and regulations and the Declaration of Helsinki. Participants were recruited starting January 6, 2022, until completion of the study in July 2022. Northwell is based in New York State, and all participants resided within the tristate area. Taking part in the study was voluntary, and participants could choose not to participate in the study or to leave the study at any time. All tests were administered at Northwell’s laboratory locations [21]. The data collected from the study were deidentified and securely transferred to researchers for analysis. All tests were provided by the Northwell laboratory testing services at no cost to the participants. To cover transportation costs, participants were compensated US $25 each time they went to a laboratory for a COVID-19 test. Participants were also allowed to keep their Fitbit device, and if they completed at least 80% of COVID-19 tests, they were entered into a random draw for a US $500 gift card.

### Algorithm Predictions Based on Health Metrics

In this study, our objective was to validate the performance of a previously developed model to detect COVID-19 using wearable physiological signals in a sample of health care workers affiliated with Northwell [9]. In this prospective study, the following physiological data were collected for each user daily using data recorded by their Fitbit watch:

- Respiration rate: The estimated mean respiration rate during deep sleep when possible and during light sleep in the case of insufficient deep sleep was assessed.
- Resting heart rate: The mean nocturnal heart rate during nonrapid eye movement sleep was assessed.
- Heart rate variability: The root mean square of successive differences in the nocturnal R-R series was assessed. It was computed in 5-minute intervals, and the median value of these individual measurements over the whole night was calculated.
- Heart rate variability (entropy): The Shannon entropy of the nocturnal R-R series was assessed. It is a nonlinear time domain measurement computed using the histogram of R-R intervals over the entire night.

Since health metrics can vary substantially between users, the algorithm used Z-scored equivalents of the aforementioned metrics. The algorithm used a matrix of 5×4 observations consisting of the 4 physiological features in the past 5 days (the day of prediction and the previous 4 days). Thus, each row of the matrix represents a day of data, while each column represents a metric. The matrix was linearly interpolated to handle missing data but only when there were data for a minimum of 3 days. Having less than 3 nights of data on a rolling window of the past 5 days was the condition where the algorithm could not generate any predictions. Then, an “image” of 28×28×1, with the last dimension indicating that there was only 1 color channel, was created by resizing each 5×4 matrix. Each image was an input for a 1-dimension convolutional stage with *m* filters. A dense layer was used to reduce the *m* convolutional features to a smaller feature set *N1*. At this stage, an array of *n* external inputs was applied, including age, gender, and BMI. The final dense layer led to a Softmax function with 2 possible output classes: positive and negative (more information on model development has been provided previously [9]).

### Model Evaluation and Statistical Analysis

**Figure 1:**
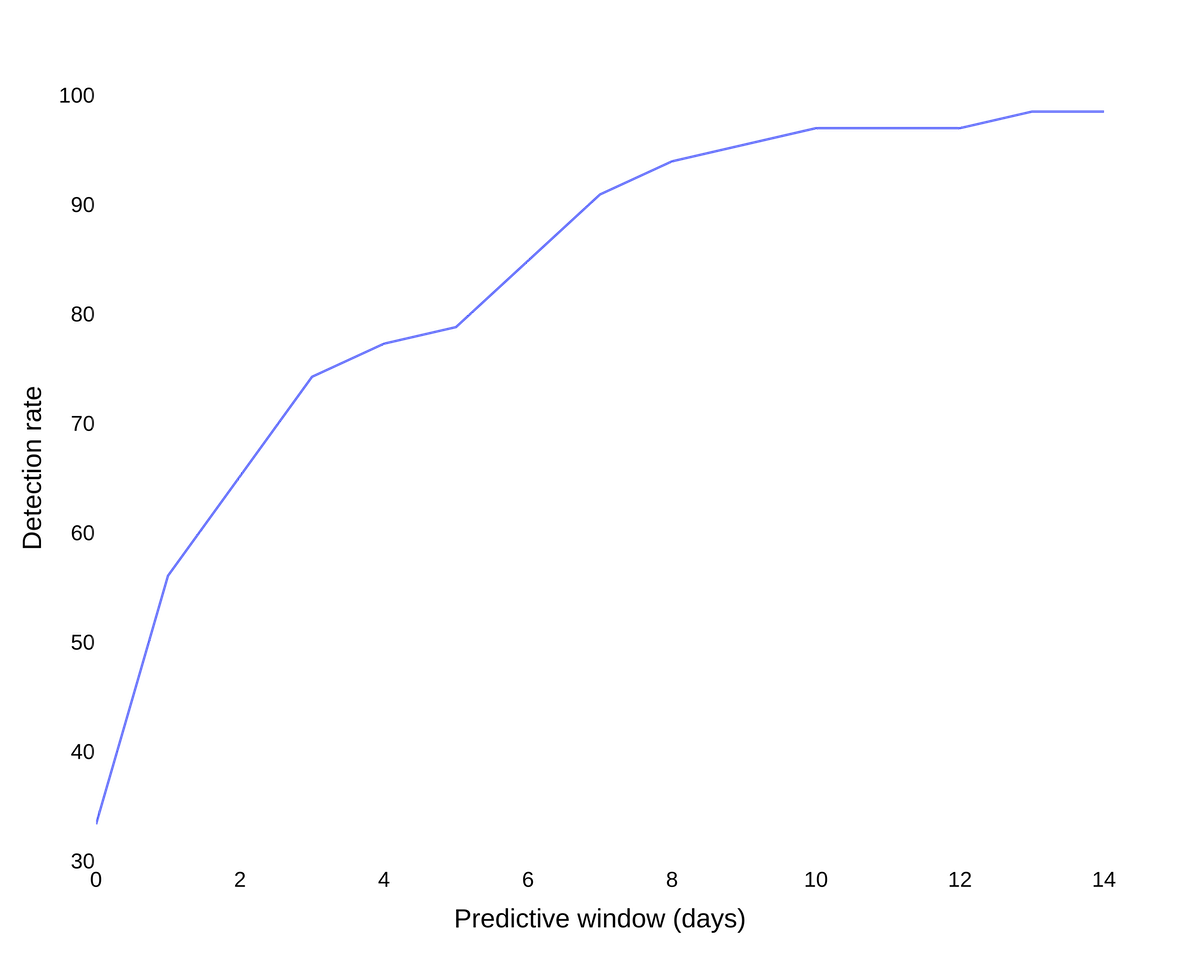
Positive algorithm detection rate of polymerase chain reaction (PCR)-confirmed COVID-19 relative to the length of the predictive window. The predictive window was the time window (days) between the test date and the date for an alert to be accepted as a correct detection (ie, predictive window=8; alert generated within 8 days prior to the test date counted as a correct detection). A wider predictive window was associated with a higher detection rate of the algorithm for PCR-confirmed COVID-19 cases.

Our model was previously developed using data collected from Fitbit users and a retrospective self-report survey of COVID-19 infections with no laboratory confirmation on the timing or accuracy of positive tests. In this study, we validated the performance of the previously developed model using data collected in a prospective study on a sample of health care workers. Participants who received a positive alert were instructed to undergo a respiratory viral panel (RVP) test to confirm any upper respiratory infections. Participants were only notified in case of positive alerts. Moreover, participants were asked to report any positive home or laboratory test results to study coordinators in cases where they got tested for reasons other than positive alerts from our study. For evaluating the algorithm performance, positive algorithm detections were defined as participants with positive test results who received an alert within 8 days prior to a positive test. The choice of 8 days as a predictive window is based on previous published work [22,23] and a sensitivity analysis of the detection rate relative to the predictive window in our study (Figure 1). In this study, our primary goal was to detect COVID-19 (ie, SARS-CoV-2 virus). In a subsequent secondary analysis, we considered different definitions of positive algorithm detections (ie, positive SARS-CoV-2 plus home test, positive respiratory viruses such as influenza, etc). The detection rate was defined as the ratio of positive algorithm detections over all positives defined based on different tests. We defined false algorithm detection as the number of participants who tested negative within 8 days after receiving a positive alert. The estimated false-positive rate was defined as the ratio of false algorithm detections over all negative alerts that did not report any positive test within the next 8 days. The positive-predictive value was defined as positive algorithm detections over all positive alerts generated. In cases where participants received positive alerts and did not act upon the alerts to get tested (ie, 153 of the 665 positive alerts, 23.0%), we assumed the test results were negative, and they were included in the denominator for the positive-predictive value.

### Study Procedure

After signing the written consent form, participants were led to an onboarding survey involving initial baseline questionnaires and collecting demographic information. Participants were prospectively issued a smartwatch (Fitbit Sense or Fitbit Versa 3) and asked to download the associated Fitbit app. After onboarding, every participant was instructed to fill out a daily questionnaire for the symptoms of COVID-19 throughout the study. Participants received a text message each morning at 7:30 AM, which included a link to a survey on the N1Thrive (Twistle) platform. The survey included questions related to COVID-19 symptoms experienced by the participant and 3 additional questions. The questions were as follows:

1. Are you currently experiencing any of the following?
  - Fever of 100 °F or feeling unusually hot (if no thermometer is available), accompanied by shivering/chills
  - Sore throat
  - New cough not related to a chronic condition
  - Runny or stuffy nose, or nasal congestion (not related to allergies)
  - Difficulty breathing or shortness of breath
  - Diarrhea unrelated to a chronic condition
  - Nausea or vomiting
  - Headache unrelated to a chronic condition
  - Fatigue unrelated to a chronic condition
  - Muscle aches unrelated to a chronic condition
  - New loss of sense of taste or smell
2. Have you had a POSITIVE COVID-19 test in the past 10 days?
3. Have you been within 6 feet for more than 15 minutes with a confirmed or suspected COVID-19 case in the past 14 days WITHOUT PROPER PPE?
4. Yesterday, how stressed were you across the entire day?

All questions involved yes or no responses, apart from the stress question, which had 5 options (relaxed, slightly stressed, moderately stressed, very stressed, or extremely stressed). If participants did not complete a daily symptom questionnaire at least 4 times in a given week, they were contacted by the study administrative team to remind them of the importance of being adherent to the study surveys. No action was taken if a person reported being exposed or being symptomatic from the study team, unless the participant received a positive alert from the Fitbit device.

The algorithm required 18 days to establish a baseline for physiological features before the generation of any prediction was possible. From day 19 onward, participants were only alerted when the algorithm generated a positive alert. This notification informed participants that their physiological measurements were outside their normal range and that they needed to contact the research team to arrange additional testing. In the case of an alert, it was sent an hour after the daily symptom survey to avoid any bias in the survey responses. However, we could not confirm whether the surveys were filled out prior to the later text. Study staff members were also alerted when participants received a positive alert, and they reached out on the same day to any participants who did not contact the study team for arrangements to get tested. Participants were instructed to undergo an RVP test at their preferred testing location (ie, across Northwell’s 21 hospitals). The RVP test was used to confirm the presence of COVID-19 or multiple upper respiratory infections. The RVP test (respiratory viral/bacterial detection panel by NAT [24]) used the multiplex amplified nucleic acid test that adopts polymerase chain reaction (PCR) for detecting influenza A virus (H1, H1-2009, and H3), influenza B virus, respiratory syncytial virus (RSV), human metapneumovirus, parainfluenza virus (types 1, 2, 3, and 4), rhinovirus/enterovirus, coronavirus (229E, HKU1, NL63, and OC43), adenovirus, *Chlamydophila pneumoniae*, *Mycoplasma pneumoniae*, and SARS-CoV-2.

**Figure 2:**
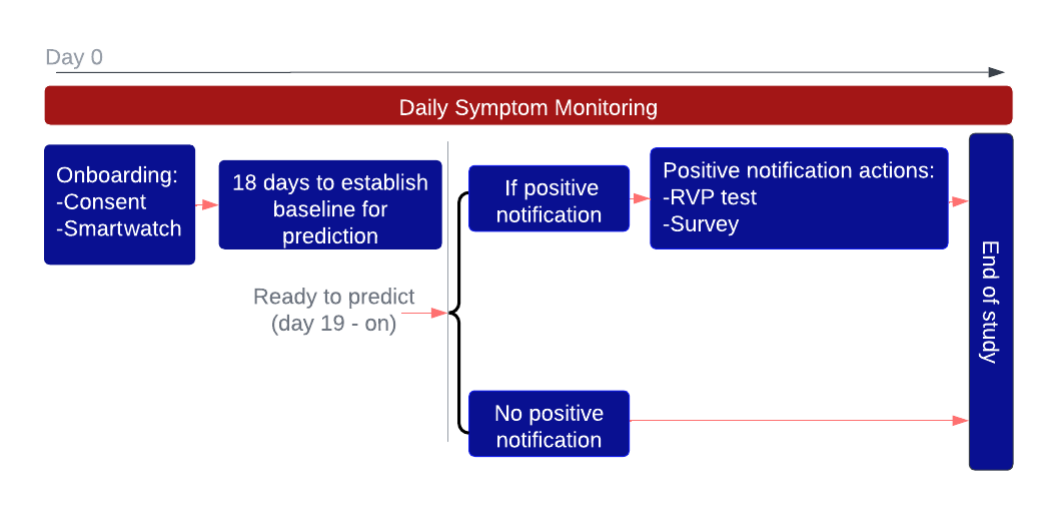
Study protocol. Day 0 to day 18: onboarding, baseline measurement, and issuing new devices. Upon receiving a positive alert from day 19 onward, participants were instructed to take the respiratory viral panel (RVP) test as well as fill out follow-up questionnaires the next day after receiving an alert. Daily symptom questionnaires were filled out throughout the study. Participants only received notifications for positive alerts. The algorithm required at least 3 nights of data in a rolling window of the past 5 nights to be able to generate predictions. In total, 89 participants in this study did not adhere to the study guidelines, and the algorithm could not generate any prediction owing to infrequent use of the smartwatch.

Figure 2 shows the study protocol. All the tests were provided by the Northwell laboratory testing service at no cost to the participants. When positive alerts were generated, both the participants and the study team at Northwell received the notifications (no notification was sent in case of a negative alert). If the study team did not hear back from the participants regarding the arrangement of getting tested on the day they were flagged, the study team followed up with them the following day and each subsequent day up to 7 days. At the close of that 7-day period after the alert, they checked to see if they had completed the test and then informed them that it was no longer necessary to go for the test but they should still complete the follow-up survey. Participants were not excluded if they did not get tested within the defined window for a positive alert since our analysis was based on intent to treat rather than per protocol. Participants were instructed to self-report any positive test results to the research team when they did not get an alert (ie, positive home tests or positive laboratory results for reasons other than positive alerts from our study).

When a participant received a positive alert from the algorithm, the alerting was suppressed for the following 5 days, regardless of the algorithm output, in order to reduce the testing burden. Participants who received a positive alert were instructed to fill out an additional questionnaire about their prior day’s behavior, including physical activity (ie, intense exercise beyond routine), stress (ie, life and work related), and the number of alcoholic drinks and amount of caffeine consumed during that period. The survey questions were as follows:

1. Overall, how do you feel about last night’s sleep quality?
2. How many alcoholic drinks did you consume yesterday?
3. How many caffeinated drinks did you consume after 12 PM yesterday?
4. Yesterday, how often did you feel at least slightly stressed?
5. Please select all that apply: I exercised yesterday, I meditated yesterday, I am currently sick, I am currently under quarantine for COVID-19, None of the above.
6. Did you change any medication or drug use in the last 2 days?
7. Did you exercise significantly more than your normal routine yesterday?
8. Did you feel like you were at your normal level of health yesterday?
9. Are there any other unusual circumstances to report from yesterday that may have been outside of your normal daily activities?

## Results

### Overview

In total, 577 participants were enrolled in this study between January 6, 2022, and July 20, 2022. Across the participants enrolled in the study, 470 successfully completed the study, 89 did not provide sufficient wearable physiological data to receive any prediction from the algorithm (ie, did not wear the watch at least 3 nights in a rolling window of the past 5 nights during the 16 weeks of the study), and 18 withdrew from the study. Participants withdrew from the study for the following reasons: change of eligibility (n=5), Fitbit issues (n=2), personal reasons (n=1), study burden (testing time commitment; n=4), Fitbit issues and study burden (n=2), and unknown (n=2). Fitbit issues included experiencing Fitbit issues and not wanting a replacement, concerns with Fitbit Ionic recall, and loss of the Fitbit device.

Table 1 presents the overall breakdown of demographics and comorbidities for all participants versus participants who tested positive for COVID-19 or other respiratory viruses over the course of the study. It is important to note that some participants tested positive more than once throughout the study, and the numbers in Table 1 refer to the numbers of participants and not the numbers of positive upper respiratory infection events. These infection events were at least more than a week apart from each other.

**Table 1:** Demographics of participants based on the study onboarding survey.

Characteristic			All participants (N=559^a^)		Participants with a positive COVID-19 test result or other upper respiratory infection (N=67^b^)
Age (years), mean (SD)			46 (13)		47 (13)
**Gender, n (%)**					
	Female	426 (76)		54 (81)	
	Male	129 (23)		13 (19)	
	Other/declined to state	4 (1)		0 (0)	
**Race, n (%)**					
	Black or African American	86 (15)		8 (12)	
	Asian	81 (14)		11 (16)	
	White	316 (56)		41 (61)	
	Other	76 (14)		7 (10)	
**Comorbidities, n (%)**					
	Anxiety	97 (17)		10 (15)	
	Depression	46 (8)		7 (10)	
	Smoking cigarettes	18 (3)		2 (3)	
	Diabetes	32 (6)		1 (1)	
	Taking insulin	7 (1)		0 (0)	
	Cardiac condition	18 (3)		2 (3)	
	Cancer condition	8 (1)		1 (1)	
	Asthma emphysema and bronchitis	63 (11)		7 (10)	
	Rheumatoid arthritis	78 (14)		9 (13)	
	Apnea condition	41 (7)		5 (7)	
	Thyroid condition	66 (12)		9 (13)	
	Medication suppressing the immune system	24 (4)		1 (1)	

^a^The number of participants who completed the study based on the onboarding survey. Of the 577 participants who were enrolled, 18 dropped out. Among the participants who completed the study, 89 did not receive any prediction owing to insufficient physiological data.

^b^Distinct users who had positive upper respiratory infection test results. Note that some participants tested positive more than once (ie, 80 upper respiratory infection events among the 67 participants).

**Figure 3:**
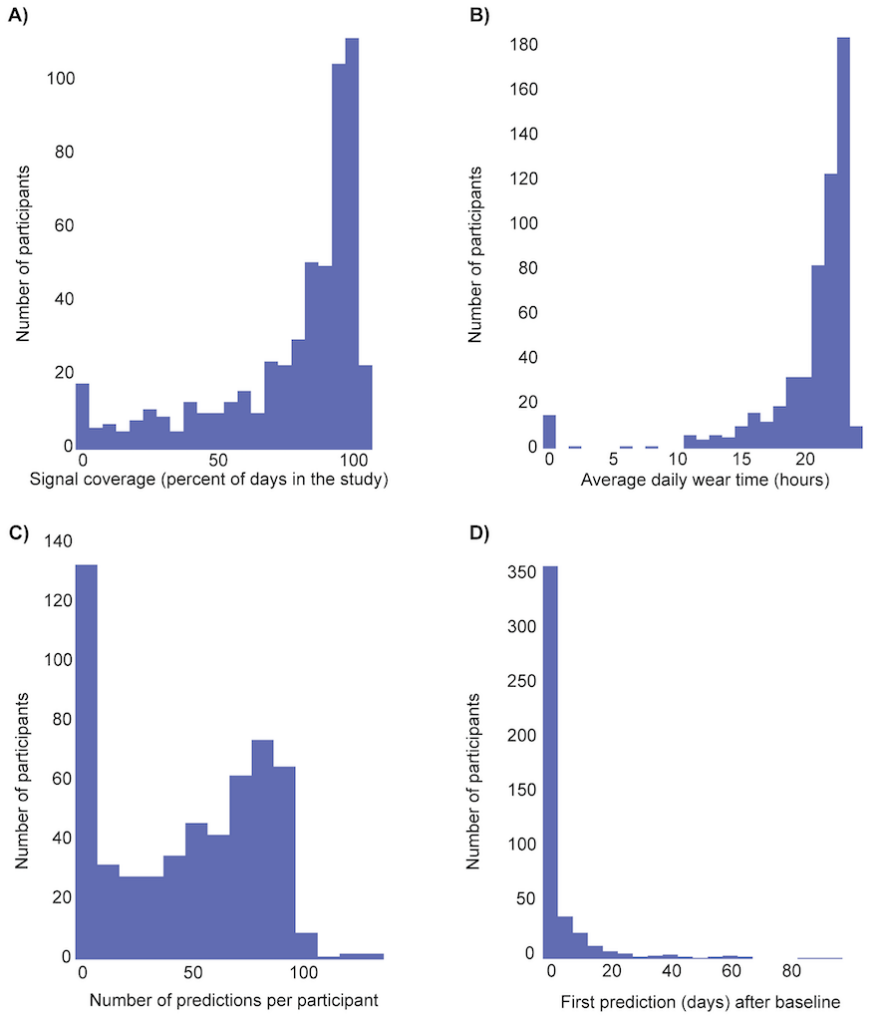
Wear time and algorithm predictions during the study. (A) Distribution of signal coverage (percentage of days participants wore the watch to bed during the study). (B) Distribution of the average daily smartwatch wear time (hours) during the study across all participants. (C) Number of predictions received by each participant during the study. (D) Distribution of time when the first algorithm prediction was generated after establishing baseline.

During the study, 81.9% (458/559) of participants wore the smartwatch to bed for more than 50% of the days in the study (Figure 3A). In terms of daily wear time, 97.0% (542/559) of participants wore the smartwatch for more than 10 hours a day (Figure 3B). Across the participants who established a baseline, 93.0% (437/470) received their first algorithm prediction (ie, positive or negative) within 20 days after baseline completion (Figure 3D).

In total, the algorithm could generate predictions for 470 participants, including both positive and negative predictions (Figure 3C). It is important to note that participants were only notified if the algorithm generated positive alerts. The algorithm generated 665 positive alerts during the study, and of these, 153 (23.0%) were not acted upon (ie, participants did not undergo an RVP test after the alert). Across the 512 alerts for which participants underwent an RVP test or home test, 63 involved confirmed cases of upper respiratory infection. Moreover, participants were instructed to report any positive upper respiratory virus test result they received during the study for reasons other than positive alerts from our algorithm to cover missed respiratory infections during the study. We received a total of 17 positive upper respiratory virus test reports from our participants without a positive alert. The breakdown was as follows: 13 positive home tests, 3 positive COVID-19 tests, and 1 positive upper respiratory virus test (ie, influenza).

In total, 31 positive PCR tests of COVID-19 and 35 positive tests of other respiratory viruses, such as influenza, adenovirus, and common cold, were collected in laboratory tests during the study. A further 14 COVID-19 cases were reported from positive home tests. The breakdown of all positive laboratory results is shown in Table 2.

**Table 2:** Breakdown of all positive upper respiratory infection tests during the study.

Virus	Positive test result (N=80), n (%)
SARS-CoV-2	31 (39)
Coronavirus (229E, HKU1, NL63, and OC43); not COVID-19	13 (16)
Enterovirus/rhinovirus	14 (18)
Adenovirus	1 (1)
Enterovirus/rhinovirus and human metapneumovirus	1 (1)
Human metapneumovirus	2 (3)
Influenza A	1 (1)
Influenza A/H3	2 (3)
Parainfluenza 3	1 (1)
COVID-19 home test	14 (18)

### Model Performance

During the study, the algorithm generated daily predictions for upper respiratory infections, including COVID-19, using wearable physiological signals. In total, 27,636 predictions were generated (positive or negative alerts) over the course of the study. It is important to note that participants only received a notification in the case of a positive alert. Based on the protocol, participants were only instructed to undergo testing after receiving a positive alert from the algorithm. We were aware of missed detections by the participant’s self-report of home or laboratory positive results when testing was performed through Northwell laboratories. Of all the alerts, 665 were positive alerts, and across these positive alerts, 512 (77.0%) were acted upon (ie, getting tested by either an RVP or home test within 8 days from the alert).

Across the 665 positive alerts, 28 were associated with a positive SARS-CoV-2 test within 8 days after the alert, 34 were associated with a positive PCR test for other upper respiratory infections, and 1 was associated with a positive COVID-19 home test (Multimedia Appendix 1) [25].

Across the 26,971 negative alerts, there were 3 reports of positive SARS-CoV-2 results, 1 report of a positive influenza A result, and 13 reports of positive home test results that did not receive an alert, and tests were performed for reasons other than receiving an alert from our algorithm.

A detailed summary of the performance of our algorithm based on different tests is shown in Table 3. When sufficient data were available, the algorithm generated daily predictions (either positive or negative). We first examined the capability of the alerting system to detect SARS-CoV-2 identified by PCR tests as our primary objective. The results shown in the detection rate column of Table 3 also include laboratory tests obtained outside of the study procedures (people underwent a test without a positive alert prompt). Of 31 cases involving positive SARS-CoV-2 laboratory PCR test results, the algorithm detected 28 (ie, 28 of the 31 instances had a positive alert within 8 days prior to the positive result), with a detection rate of 90%. The estimated false-positive rate of the algorithm based on prediction per day was 2%; however, the positive-predictive value was low (4%). Most of the algorithm alerts could be attributed to events other than COVID-19, thereby highlighting confounders that change physiological signals and subsequently trigger the algorithm. If we expand the definition of positive to be either a laboratory-confirmed PCR result or a self-report home test result, 29 out of 45 instances would be detected. Only 1 instance of a home test was detected by the algorithm. There is uncertainty around the date of home test self-reports, which could contribute to the poor performance of the algorithm. In total, 62 instances of positive respiratory virus PCR tests (ie, SARS-CoV-2 or other respiratory viruses) out of 66 instances of positive SARS-CoV-2 and other respiratory virus PCR tests received an alert within 8 days prior to a positive test result. Overall, across respiratory viruses (PCR or home tests), 63 instances were detected out of 80 instances (Table 3). In Table 3, we have used the term “estimated” false-positive rate as there was a small possibility that some infections were missed (if there was no alert and they never received a test). Moreover, there was a small risk of positive infections being missed owing to the limited sensitivity of the viral test panels.

**Table 3:** Algorithm performance across different upper respiratory infection tests.

Test type	Detection rate (number detected/number tested positive; tests in the protocol and out of the protocol)	Estimated false-positive rate	Positive-predictive value
SARS-CoV-2	0.90 (28/31)	0.02 (637/26,759)	0.04 (28/665)
SARS-CoV (laboratory PCR^a^ or home test)	0.64 (29/45)	0.02 (636/26,745)	0.04 (29/665)
Respiratory viral panel test	0.94 (62/66)	0.02 (603/26,724)	0.09 (62/665)
Respiratory viral panel test and home test	0.79 (63/80)	0.02 (602/26,710)	0.09 (63/665)

^a^PCR: polymerase chain reaction.

**Figure 4:**
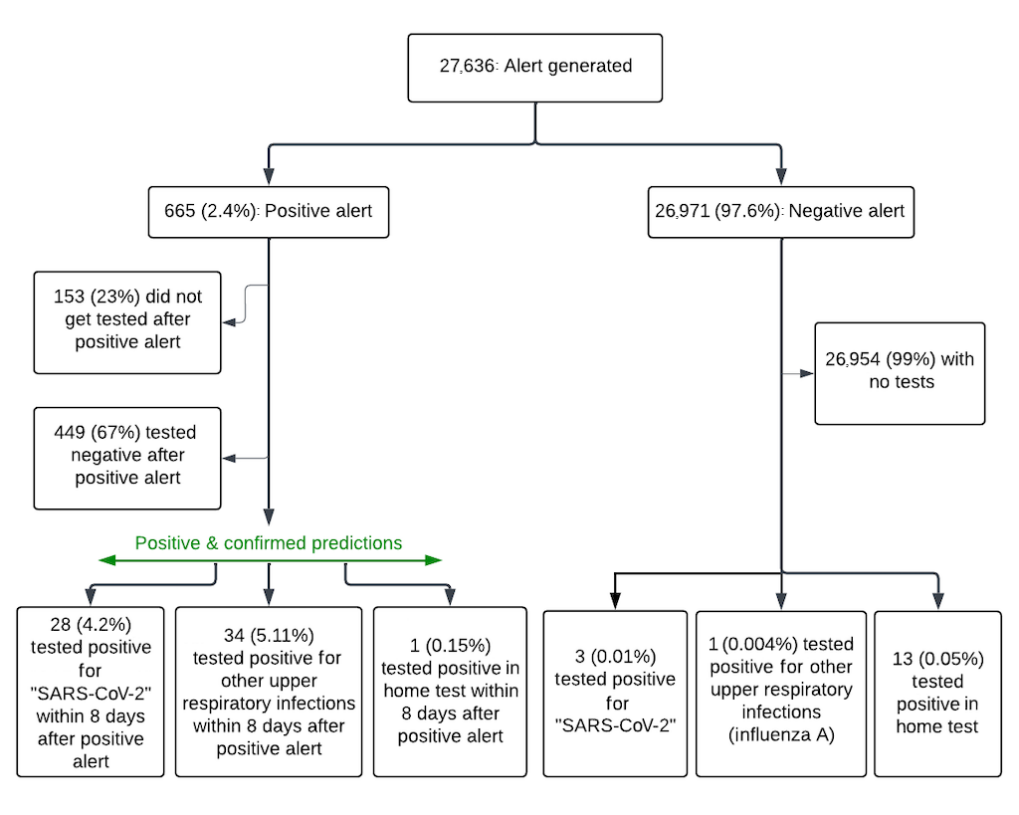
Algorithm alert flowchart following the STROBE (Strengthening the Reporting of Observational Studies in Epidemiology) reporting guidelines. Participants only received a notification to undergo testing if the algorithm generated a positive alert. Participants were instructed to report any positive upper respiratory infection test results they received for reasons other than positive alerts from our algorithm.

Another important factor for model performance evaluation was the number of false alerts each person received during the study, since this would impact the feasibility of the algorithm in real-world deployment. The distribution of false alerts is shown in Figure 4. A false alert was defined as a positive alert without a positive test result (COVID-19 or other respiratory viruses confirmed with a PCR or home test) within 8 days after the alert. Overall, out of 665 positive alerts across 470 participants, 172 participants never received a false alert (Figure 4). The maximum number of false alerts for a single participant was 8 (out of 102 predictions). The reason for this large number of false alerts is unclear. For the analysis, positive alerts that participants did not act upon (ie, 23% of positive alerts) were considered as negative test results and were counted toward the false alerts. The number of false alerts could be less in reality.

### Symptoms

Many strategies for managing COVID-19 as a public health issue rely on self-reporting of symptoms (followed by voluntary self-isolation), and symptoms can help flag the disease if present [26,27]. In real-time alerting systems that rely solely on physiological signals, one of the factors limiting model performance is confounding events (ie, physical or emotional stress) [11]. Daily symptom tracking could be a potential candidate that can bring context to alerting systems and potentially increase the sensitivity or specificity of these algorithms. In this study, we tracked daily symptoms as a secondary objective to evaluate the most commonly reported symptoms and the percentage of participants who reported symptoms in positive-detection cases versus false-positive cases.

The most commonly reported symptoms in positive respiratory virus infection tests across participants were “runny or stuffy nose, or nasal congestion (not related to allergies),” “fatigue not related to a chronic condition,” “cough,” and “sore throat,” which are in line with the findings in the literature [26,28,29]. Symptoms, such as “new loss of sense of taste or smell,” “nausea or vomiting,” “difficulty breathing or shortness of breath,” and “diarrhea unrelated to a chronic condition,” had the lowest reports in our cohort with positive test results, and these findings are in line with the findings of previous reports [27].

**Figure 5:**
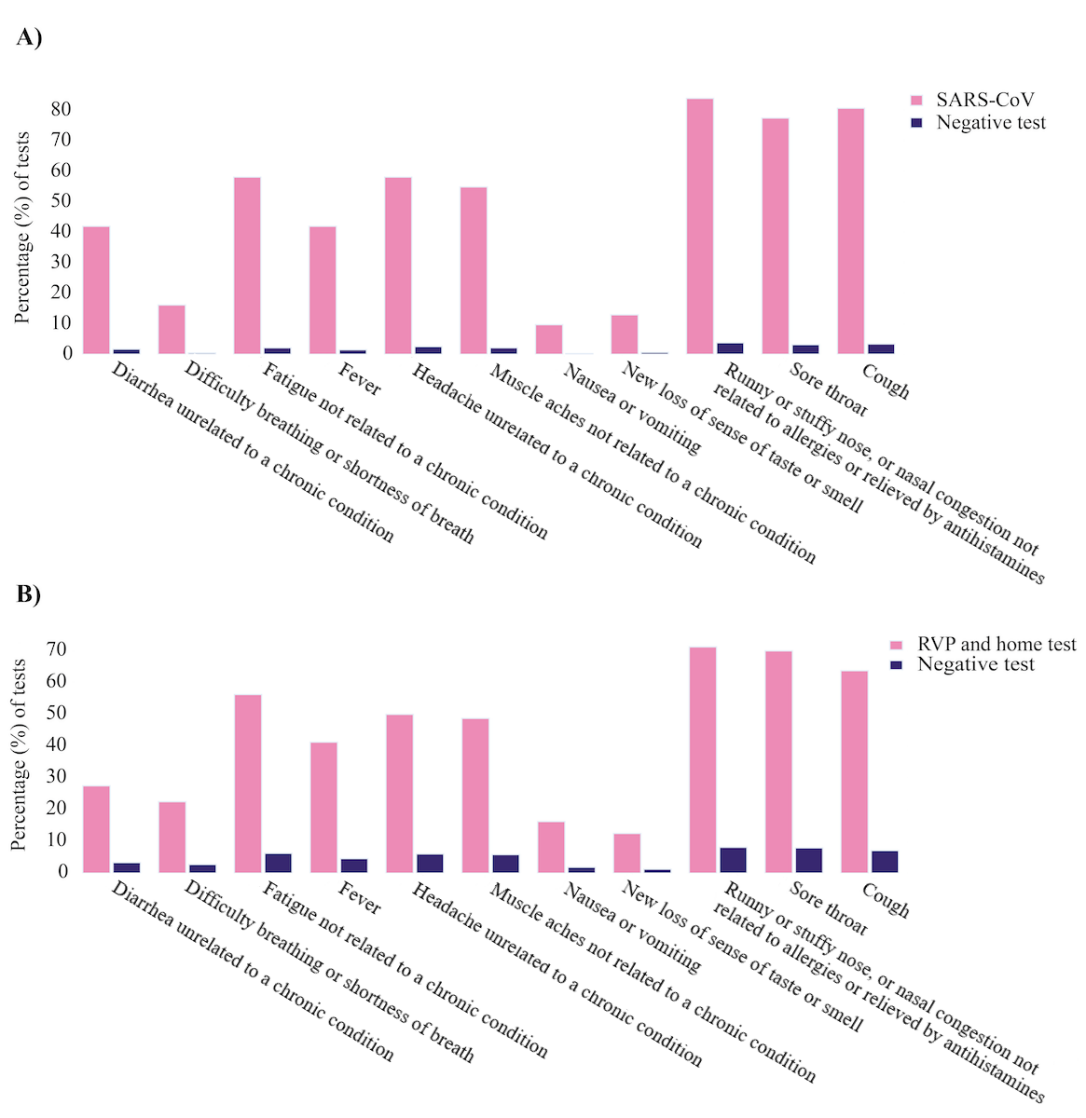
Associations of test results with symptoms across participants with positive and negative test results who received an alert. Bar plots show the percentage of positive tests associated with each symptom. Symptoms were considered within a window of 10 days before the positive test result to 10 days after the test result. (A) Participants with positive COVID-19 polymerase chain reaction (PCR) test results. (B) Participants with positive results for respiratory viruses confirmed with PCR or home tests versus participants who received an alert with negative PCR test results. RVP: respiratory viral panel.

We observed a significant difference in symptom reports across participants with positive SARS-CoV-2 PCR test results versus participants with negative PCR test results within a 20-day window centered around the test date (ie, 10 days before the positive test result to 10 days after the test result) (Figure 5). Among all participants who had a positive SARS-CoV-2 PCR test result, approximately 80% (25/31, 81%) reported “cough,” “sore throat,” or “runny or stuffy nose, or nasal congestion not related to allergies or relieved by antihistamines” within a 20-day window centered around the test date (ie, 10 days before the positive test result to 10 days after the test result) (Figure 5A). Among participants with positive results of COVID-19 or other respiratory viruses confirmed with either PCR or home tests, 70% (56/80) reported “runny or stuffy nose, or nasal congestion not related to allergies or relieved by antihistamines,” “cough,” or “sore throat” within a 20-day window centered around the test date (Figure 5B).

### Survey on the Next Day After Receiving an Alert

After receiving an alert from the algorithm, participants were asked to fill out a follow-up questionnaire to investigate potential confounding factors. In total, we received 569 completed surveys the next day after receiving an alert out of 665 generated alerts (85% completion rate for the follow-up survey). Table 4 shows the percentage of participants who reported each of the conditions. In total, across all 569 participants who filled out the questionnaire after receiving an alert, 362 (63.6%) reported a reason related to a physical or emotional stress event, including COVID-19.

**Table 4:** Association of algorithm alerts with self-reported physical or emotional stress events.

Physical or emotional event	Alerts associated with each event (N=569), n (%)
COVID-19 and other upper respiratory infections^a^	63 (11.1)
Other sickness^b^	28 (4.9)
Stress^c^	91 (16.0)
Poor sleep^d^	78 (13.7)
Intense exercise^e^	55 (9.7)
Alcohol consumption^f^	25 (4.4)
Pain^g^	13 (2.3)
Caffeine consumption^h^	9 (1.6)
No reason	207 (36.4)

^a^COVID-19 and other upper respiratory infections were defined as all upper respiratory viruses detected in our study by the respiratory viral panel test (eg, COVID-19 and influenza).

^b^Other sickness was defined as sickness other than upper respiratory infections reported by participants (ie, stomach bug, COVID-19 booster, shingles vaccine, seasonal allergy, recovery from surgery, eye infection, gallstone, etc).

^c^Stress was defined as a stress score of “4” (fairly often) or “5” (very often).

^d^Poor sleep was defined as a sleep score of “1” (poor sleep).

^e^Intense exercise was defined as exercising significantly more than normal.

^f^Alcohol consumption was defined as the consumption of more than 2 glasses of alcoholic drinks.

^g^Pain was defined as reporting strong pain (eg, menstrual cramps, pain after knee replacement surgery, etc).

^h^Caffeine consumption was defined as the consumption of more than 2 cups of coffee after 12 PM.

### Comorbidities and Respiratory Virus Detections

Although most people who contract COVID-19 have few symptoms or become mild to moderately ill, a substantial minority are at high risk of more severe disease and adverse outcomes, including death and long COVID. This is particularly true for people with comorbidities [30]. Table 5 shows the percentage of participants with comorbidities who tested positive for COVID-19 or other respiratory viruses during this study. We also present the relative risk of testing positive in each comorbidity group with 95% CIs. None of the listed comorbidities showed a significant relative risk (Table 5).

**Table 5:** Association of comorbidities in participants who contracted COVID-19 or other respiratory viruses.

Variable	Participants (N=559), n	Positive RVP^a^ test (PCR^b^ or home test), n (%)	Relative risk of a positive RVP test result, value (95% CI)
Smoking cigarettes	18	2 (11)	0.92 (0.21-3.90)
Medication suppressing the immune system	24	1 (4)	0.32 (0.04-2.32)
Diabetes	34	1 (3)	0.22 (0.03-1.60)
Rheumatoid arthritis	78	9 (12)	0.96 (0.50-1.83)
Cancer condition	8	1 (13)	1.05 (0.13-8.39)
Asthma emphysema and bronchitis	63	7 (11)	0.92 (0.44-1.93)
Apnea condition	41	5 (12)	1.02 (0.41-2.51)
Taking beta-blockers	31	3 (10)	0.79 (0.24-2.52)
Taking antidepressants	62	8 (13)	1.10 (0.54-2.18)

^a^RVP: respiratory viral panel.

^b^PCR: polymerase chain reaction.

## Discussion

Infection detection algorithms based on wearable longitudinal physiological data provide unique opportunities for the early detection of respiratory illnesses. This technology may potentially be helpful in supporting procedures to limit the spread of infectious viruses. In this prospective study, we evaluated the performance of our previously developed alerting system for detecting COVID-19 as the primary goal and expanded the scope to detecting other upper respiratory viruses such as influenza. The model performance was evaluated on a sample of health care workers affiliated with Northwell Health in New York, which was a separate data set from the one used in the model development phase to validate the performance of the model on a population with different COVID-19 prevalences [31]. In health care workers who are at increased risk of infection and transmission of the virus, these infection detection algorithms are highly important to identify who might be at increased risk and limit the spread [32]. The observed algorithm detection rate was 90% for detecting COVID-19 cases confirmed with PCR tests and 79% for COVID-19 or other respiratory viruses confirmed with either PCR or home tests.

To limit the spread of COVID-19, it is critical to understand the symptoms. In this study, we investigated the association of symptoms across participants with positive RVP test results versus participants with negative test results. Over 80% (25/31, 81%) of participants with COVID-19 reported at least one symptom within a 20-day window centered around the test date, whereas less than 10% (40/449, 8.9%) of participants with a negative PCR test result reported a symptom within the same time window. The top 3 reported symptoms were “runny or stuffy nose, or nasal congestion,” “cough,” and “sore throat,” in line with previous reports [26,27,29]. The use of symptoms alone for infection detection is likely to limit the early and accurate detection of COVID-19 or other respiratory infectious diseases. The poor diagnostic accuracy of COVID-19 based on symptoms alone [26] stresses the importance of algorithm detection using longitudinal wearable signals for limiting the spread of viral infections. Pairing algorithm detection with symptom tracking could lead to increased performance of COVID-19 or respiratory viral infection detection.

With regard to model performance, the false-positive rate based on prediction per day was 2%, and the positive-predictive value ranged from 4% to 10% in this specific population, with an observed incidence rate of 198 cases per week per 100,000. The design of the study did not allow the calculation of the negative-predictive value. Many of the alerts generated in this study were not associated with COVID-19 or any other respiratory viruses, which is in line with the findings of previous studies [11]. This highlights the confounding factors, namely physical and emotional stress events, that could generate false alerts. Across all the generated alerts in this study, 11.1% (63/569) were related to COVID-19 or other upper respiratory infections and 4.9% (28/569) were due to other illnesses such as allergies, stomach bugs, recovery from surgery, and eye infection. The rest of the alerts were associated with stress (91/569, 16.0%), poor sleep (78/569, 13.7%), physical stress (ie, intense exercise beyond normal routine) (55/569, 9.7%), excessive caffeine or alcohol consumption (34/569, 6.0%), and pain (13/569, 2.3%). Based on participant questionnaires, in 47.6% (271/569) of the generated alerts, the alerts could be easily self-contextualized by the participants due to the aforementioned physical and emotional stress events and might lead to not undergoing any tests.

This study had several limitations. For participants who did not receive a positive alert, we relied on self-report test results to identify cases where the algorithm missed a detection (ie, false-negative cases). It is possible that asymptomatic cases of COVID-19 were missed throughout the study owing to a lack of active COVID-19 surveillance. Continuous testing would provide a better evaluation of model performance. Other limitations of longitudinal wearable studies are adherence to study guidelines and adherence to wearing the watch frequently to provide enough data for prediction. In this study, 89 participants did not have sufficient data to generate any prediction through the study. In terms of the completion rate, 77% mentioned undergoing a test after a positive alert and 85% filled out the follow-up questionnaire for confounding events. To further investigate confounding events (ie, physical or emotional stress), it is recommended to conduct a daily survey of physical or emotional stress events to better estimate the association of alerts with these events and eliminate the placebo effect related to receiving a survey after a positive alert.

With increasingly sophisticated sensors and the ability to add brief contextual questions about behaviors, it is reasonable to expect an increase in the performance of the algorithm and a reduction in false alerts generated due to confounding factors such as stress, alcohol consumption, and exercise. Moreover, including contextual information about the prevalence of the infectious disease in each region could potentially increase the model performance. With the continuous development of wearable technology and underlying algorithms, platforms that employ a variety of physiological signals can be important in the fight against infectious diseases.

## Appendix

Multimedia Appendix 1 Distribution of false alerts per participant during the study. A false alert was considered a positive alert without a positive test result (COVID-19 or other respiratory viruses confirmed with a polymerase chain reaction or home test) within 8 days after the alert. Out of 470 participants, 172 received no false alerts and 137 received 1 false alert.

## Abbreviations

PCR: polymerase chain reaction
RVP: respiratory viral panel

## Data Availability

The data sets presented in this article are not readily available because Fitbit’s privacy policy does not permit us to make the raw data available to third parties, including researchers, outside of our web API Oauth 2.0 consent process. For specific questions, contact Fitbit [33]. The data sets analyzed during the study are available upon reasonable request from the corresponding author.
