# Supplementary figures and images for: Detection of Common Respiratory Infections, Including COVID-19, Using Consumer Wearable Devices in Health Care Workers: Prospective Model Validation Study
Source: JMIR Form Res. 2024 Jul 17;8:e53716. doi: 10.2196/53716 (PMC11292157; doi:10.2196/53716)

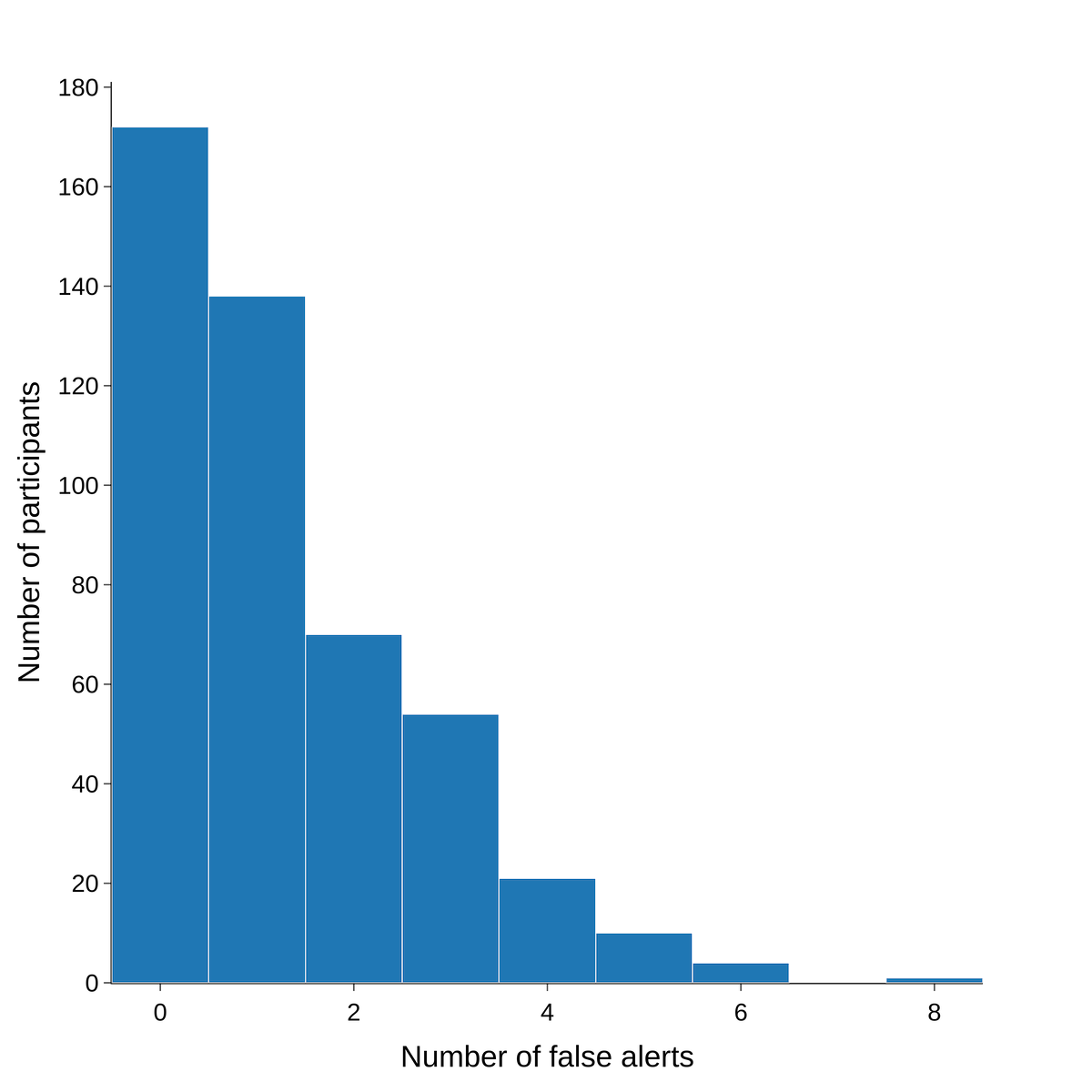

Supplement: Multimedia Appendix 1 [file formative_v8i1e53716_app1.png]
